# Case Report: Compound Heterozygous Variants of *SLC13A3* Identified in a Chinese Patient With Acute Reversible Leukoencephalopathy and α-Ketoglutarate Accumulation

**DOI:** 10.3389/fped.2021.801719

**Published:** 2021-12-13

**Authors:** Qingyun Kang, Liming Yang, Hongmei Liao, Sai Yang, Haiyang Yang, Zeshu Ning, Caishi Liao, Liwen Wu

**Affiliations:** Department of Neurology, Hunan Children's Hospital, Changsha, China

**Keywords:** *SLC13A3*, leukoencephalopathy, α-ketoglutarate, NaDC3, case report

## Abstract

**Background:** *SLC13A3* gene encodes the Na^+^/dicarboxylate cotransporter 3 (NaDC3), which locates on the plasma membrane and is mainly expressed in kidney, astrocytes and the choroid plexus. It imports four to six carbon dicarboxylates together with three Na^+^ ions into the cytosol. Nowadays, pathogenic variants of *SLC13A3* gene were found to cause acute reversible leukoencephalopathy and α-ketoglutarate accumulation (ARLIAK) in patients. Here, we report two novel *SLC13A3* variants c.185C>T (p.T62M) and c.331C>T (p.R111^*^) identified in a Chinese patient with ARLIAK.

**Case Presentation:** The patient was a Chinese girl aged 13 years and 7 months old, who had acute, recurrent neurological deterioration during two febrile episodes. She presented with reversible leukoencephalopathy and increased urinary excretion of α-ketoglutarate. Genetic studies revealed compound heterozygous variants (c.185C>T, p.T62M, and c.331C>T, p.R111^*^) in *SLC13A3*, which had not been reported previously.

**Conclusions:** These findings expand the variant spectrum of *SLC13A3*, providing the basis for the further study of this rare disease.

## Introduction

The *SLC13A3* gene encodes the Na^+^/dicarboxylate cotransporter 3 (NaDC3), which locates on the plasma membrane and transports important metabolic intermediates into cells (1–3). Besides intermediates of the citric acid cycle, such as succinate and α-ketoglutarate (4), NaDC3 also transports other important metabolic compounds into the cell, including glutathione (5), mercaptosuccinate, and N-acetylaspartate (NAA) (6). NaDC3 plays a vital role in cell nutrition and detoxification. Pathogenic variants of *SLC13A3* lead to acute reversible leukoencephalopathy (a heterogeneous group of conditions characterized by developmental abnormalities or degeneration of white matter) and α-ketoglutarate accumulation (ARLIAK) (7). This disease is fairly rare. Only two patients have been reported previously.

Here, we report on a Chinese patient who developed acute, recurrent neurological deterioration during two febrile episodes. She had eversible leukoencephalopathy, which is caused by a novel compound heterozygous variants in *SLC13A3* (c.185C>T, p.T62M, and c.331C>T, p.R111^*^). The new case with novel *SLC13A3* reported here provides the potential for a better understanding of the phenotypic and genotypic spectra of *SLC13A3*.

## Methods

### Ethics Approval and Consent to Participate

This study was approved by the ethics committee of Hunan Children's Hospital. Written informed consent was obtained from the proband's parents with the agreement to share the clinical and genetic information for research analysis.

### Genetic Analysis

Blood samples were sent to Running Gene Inc. (Beijing, China) for the whole-exome sequencing (WES). The standard process is available in a previous report (8). And the discovered candidate causal genes were confirmed *via* Sanger sequencing.

## Results

### Case Presentation

A 13-years-7-months-old Chinese girl was adopted shortly after birth, who showed normal growth and development. At 3 years and 5 months old, the patient presented with febrile convulsion. The brain magnetic resonance imaging (MRI) and electroencephalogram (EEG) were performed, the results of which were normal.

**Figure 1:**
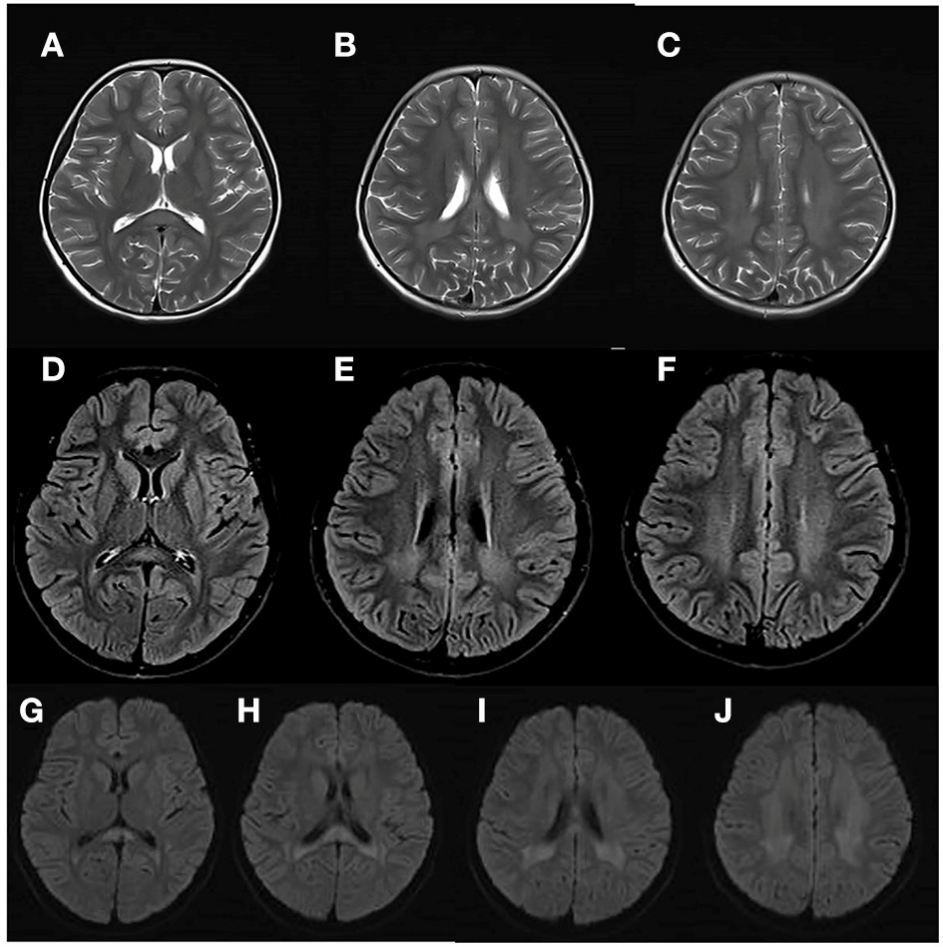
Brain MRI of the patient at her first episode of disease, at 8 years and 9 months. The white matter (WM) showed hyperintense in the T2-weighted axial **(A–C)** and FLAIR axial **(D–F)** views, and restricted diffusion in the diffusion-weighted imaging **(G–J)**.

**Figure 2:**
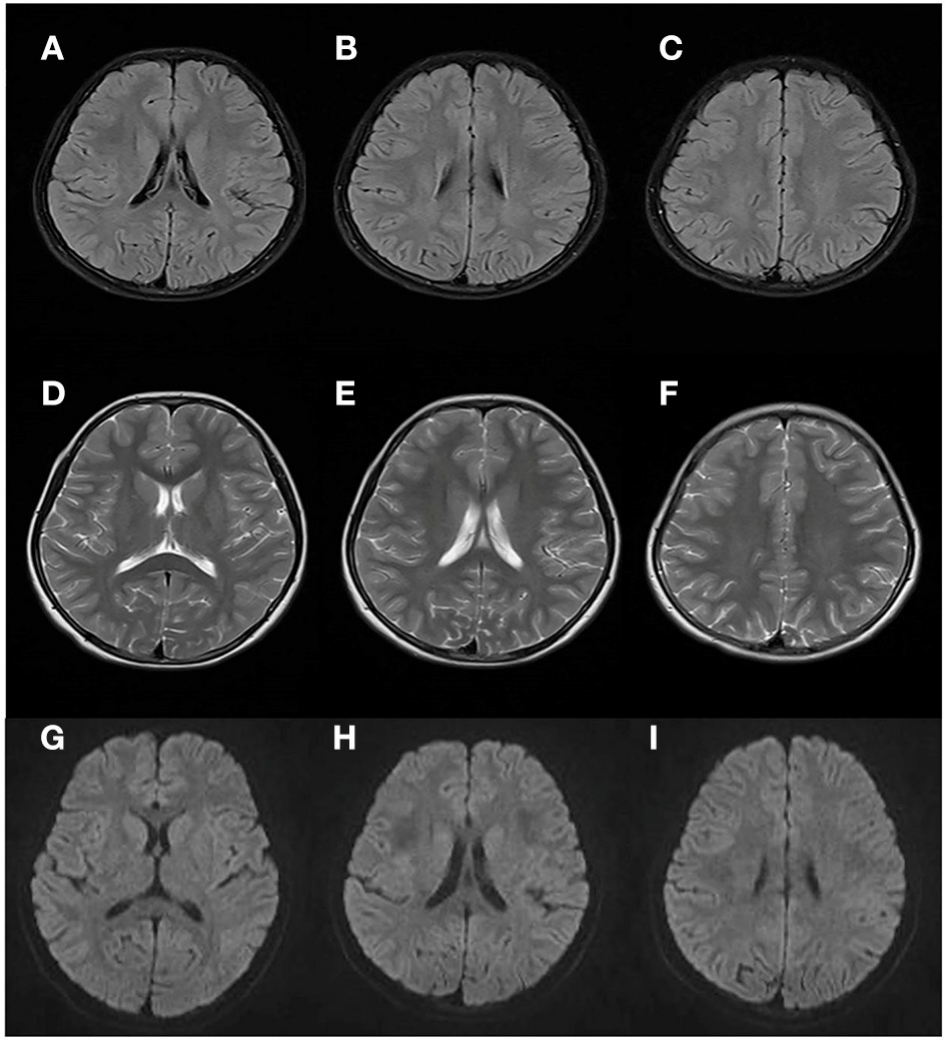
Brain MRI performed 45 days after the first attack. Results showed almost complete regression of the WM abnormalities (compared with Figure 1).

At 8 years and 9 months old, the patient developed a febrile respiratory infection. She was treated with amoxicillin granules, but the treatment did not work. Two days later, the fever persisted at 39.3°C and the patient showed an acute neurological deterioration including drowsiness, agitation and ataxia. A series of auxiliary tests were carried out after she was admitted to our department. Laboratory parameters, including blood glucose, electrolytes, ammonia, blood cell count, toxic and drug screen, liver and kidney function tests, were normal. A semi-quantitative urine ketones body test was positive (2+). Normal cell count and glucose were found in cerebrospinal fluid (CSF) analysis, with no elevated protein concentration and oligoclonal band. Extensive viral and bacterial testing ruled out the infectious meningoencephalitis. Brain MRI showed bilateral, symmetric signal abnormalities in the white matter (WM) of the centrum semiovale and the periventricular regions, signal abnormalities in the splenium of the corpus callosum, and the head of the caudate nucleus (Figure 1). The WM showed hyperintense in the T2-weighted axial (Figures 1A–C) and FLAIR axial (Figures 1D–F) views, and restricted diffusion in the diffusion-weighted imaging (Figures 1G–J). Brain magnetic resonance spectroscopy (MRS) was performed, the result of which showed normal lactate and NAA peaks. No other urine test abnormalities were observed, except for an increased urinary excretion of α-ketoglutaric acid. The acyclovir and intravenous cefotaxime sodium were administered to treat the suspected intracranial infection. After a few days, the patient returned to an almost complete normal clinical status. Brain MRI was performed 45 days later, and the WM abnormalities were almost completely subsided (Figures 2A–I).

**Figure 3:**
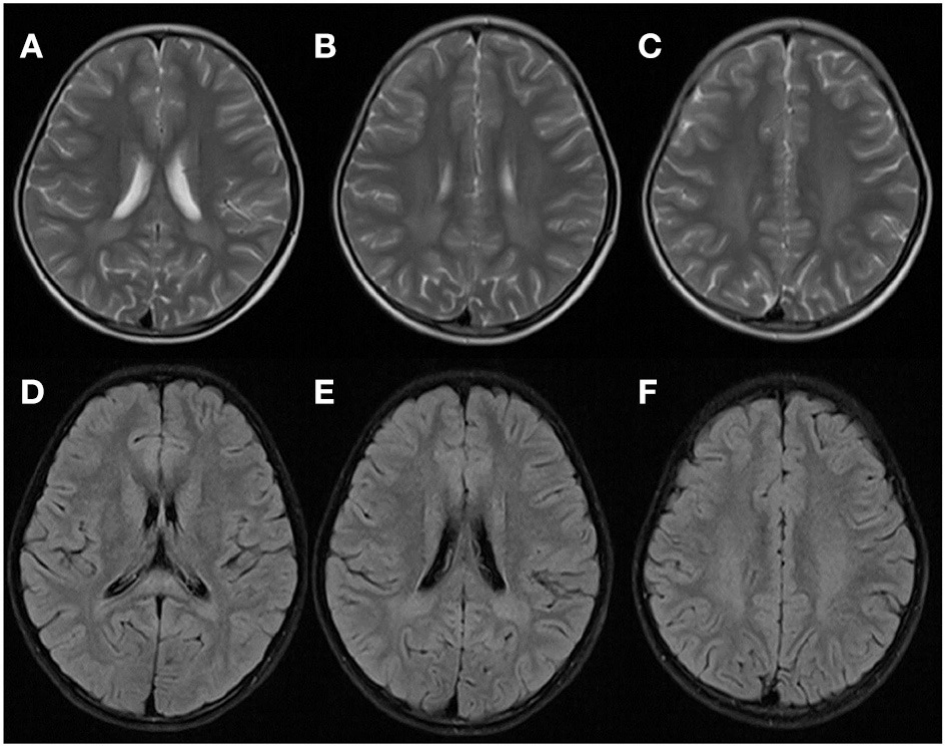
Brain MRI of the patient at the second episode of disease 2 years later. WM appeared hyperintense in T2-weighted axial **(A–C)** and FLAIR axial **(D–F)** views, which was similar with that observed during the first attack.

Two years later, after experiencing a fever (38.8°C) from tonsillitis for 2 days, the patient presented with an acute neurological dysfunction including drowsiness, agitation, dizziness and fatigue. She started intravenous cefotaxime sodium and acyclovir. CT scans of the brain were normal. The brain MRI of WM in the T2-weighted axial and FLAIR axial views were similar to that observed in the first acute episode (Figure 3). Results of CSF analysis, including protein, glucose, cell count, and oligoclonal band were normal results. While an increase in urinary excretion of α-ketoglutaric acid was observed in the urine again. She recovered within 2 days.

Our patient was seen in the outpatient clinic 1 month, 1 year, and 3 years after the second acute event with satisfactory clinical follow-up. The clinical status and examination results were normal.

### Genetic Analysis

**Figure 4:**
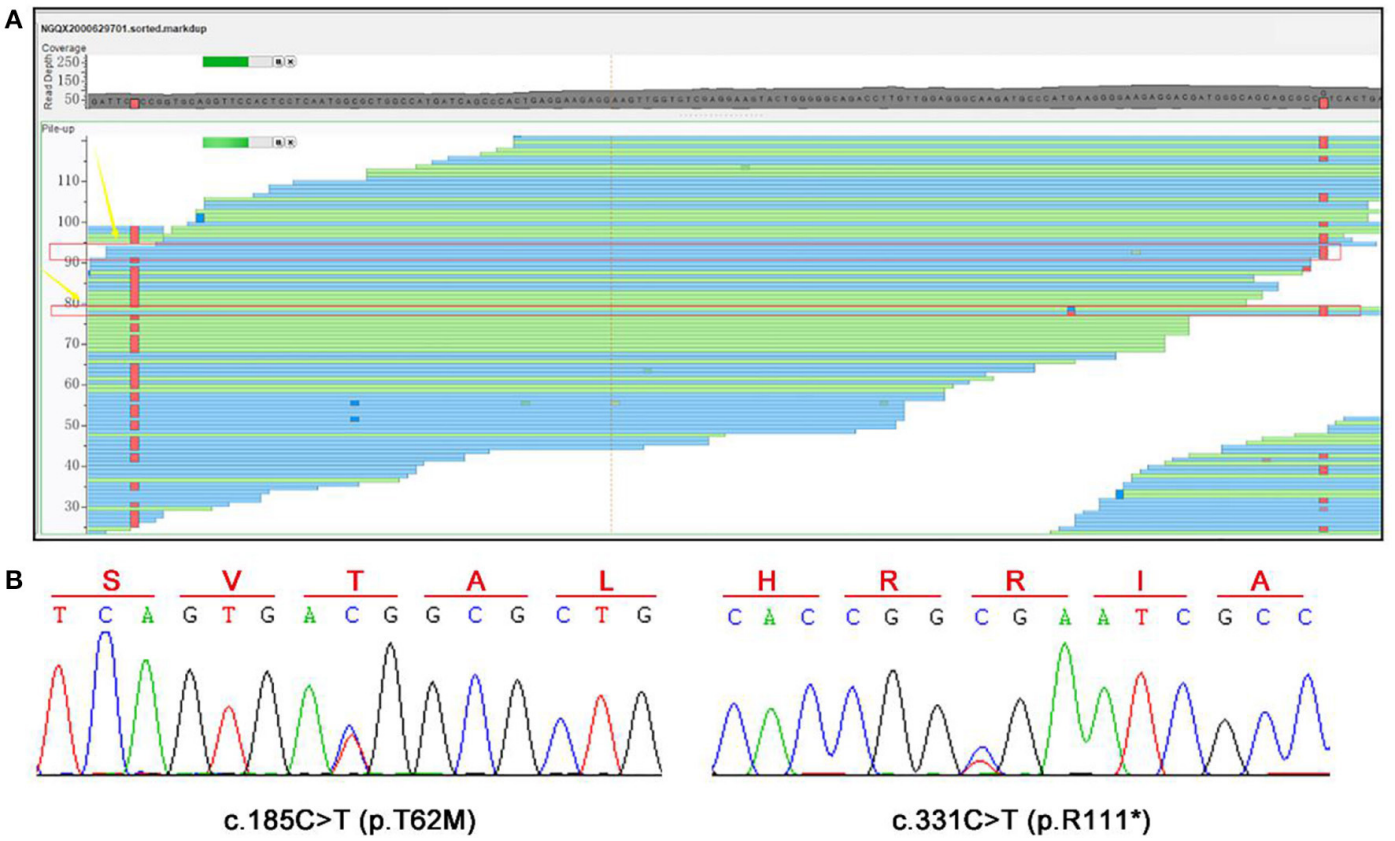
WES and Sanger sequencing results. **(A)** A compound heterozygous variants of *SLC13A3*, c.185C>T (p.T62M), and c.331C>T (p.R111*), were identified. **(B)** Sanger sequencing was performed to confirm these two variants.

In our case, a compound heterozygous variants of *SLC13A3* were identified. The compound heterozygous c.185C>T and c.331C>T (Figures 4A,B) resulted in a substitution (p.T62M) and a stop codon (p.R111^*^) in the protein, respectively. Although the biological parents could not be obtained, we confirmed that the two variants were inherited from the mother and father, respectively, as analyzed from the BAM files of the second-generation sequencing (Figure 4A). So far, these two variants have not been reported in any databases.

## Discussion and Conclusion

In 2019, Dewulf et al. reported two patients with biallelic *SLC13A3* variants. One patient presented with drowsiness, ataxia, and dysarthria after a febrile respiratory tract infection, which indicated an acute neurological deterioration (7). The other patient presented with drowsiness, poor contact, dysarthria, peripheral motor abnormalities, and global hypotonia after febrile tonsillitis, and relapsed 6 years later after febrile respiratory tract infection. They both returned to an almost completely normal clinical status after a short period of supportive treatment. It was the first report showing that the biallelic variants of *SLC13A3* are associated with reversible leukoencephalopathy and α-ketoglutarate accumulation. No other cases have been reported until now.

Compound heterozygous variants c.185C>T (p.T62M) and c.331C>T (p.R111^*^) of *SLC13A3* were identified in our patient, whose clinical presentations were consistent with that reported by Dewulf et al.. The patient presented with acute neurological deterioration after a febrile respiratory infection, symptoms including drowsiness, agitation, and ataxia. A few days later, she recovered almost completely. Two years pass, the patient relapsed after a febrile tonsillitis. After a short period of supportive treatment, she recovered as before. Like other central nervous system diseases caused by inborn errors of metabolism, triggering events such as fever seem to be necessary to cause lesions (9). How febrile disorders ultimately lead to the development of loss of compensation and lesions is unclear. However, in NaDC3-deficient patients, it may be advisable to suggest controlling body temperature by appropriate measures to avoid catabolic states. The brain MRI may be an important indicator to diagnose the ARLIAK, as all three patients had similar changes in Brain MRI (Figure 1).

The *SLC13A3* gene encodes NaDC3, a plasma membrane cotransporter expressed in kidney, brain, liver, placenta, and eye (3, 10–12). It transports intermediates of the citric acid cycle (succinate and α-ketoglutarate) and other important metabolic compounds (glutathione, mercaptosuccinate, and NAA) into the cell, playing a vital role in cell nutrition, and detoxification (4–7). Pathogenic variants in SLC13A3 can cause a marked decrease in α-ketoglutarate, succinate, and NAA transport capacity, indicating a loss-of-function mechanism, and the abnormal findings in the central nervous system (CNS) could be explained (7). In the kidney, NaDC3 is detected in the luminal membrane, reabsorbing dicarboxylates from the glomerular filtrate (13). The decrease in NaDC3 function may be responsible for the accumulation of α-ketoglutarate in the urine. In our patient, the missense variant c.185C>T resulted in a substitution (p.T62M), which amino acid locates in the second transmembrane domain. And the nonsense variant c.331C>T introduces a stop codon, which leads to a truncated protein with only 110 amino acids. Both variants could disrupt the structure and function of NaDC3, causing disease in the patient as a result. What should also be mentioned is that the studies on NaDC3 are limited. The exact mechanism of acute reversible leukoencephalopathy and accumulation of α-ketoglutarate caused by pathogenic *SLC13A3* variants needs more evidence to be confirmed.

SLC13A3-related ARLIAK is quitely rare, with only three patients (including our patient) reported. Because of the induction by febrile illness and the rapid return to an almost completely normal clinical state, patients may be misdiagnosed with infectious encephalitis or autoimmune encephalitis. Therefore, patients with acute reversible leukoencephalopathy of unknown origin should be screened for *SLC13A3* variants and urinary organic acids, which is beneficial for the correct diagnosis and treatment of patients. With more cases reported with novel *SLC13A3* variants, we can expand our understanding of the *SLC13A3* variants, laying the groundwork for future studies.

## Data Availability Statement

The original contributions presented in the study are included in the article/supplementary materials, further inquiries can be directed to the corresponding authors.

## Ethics Statement

Written informed consent was obtained from the individual(s), and minor(s)' legal guardian/next of kin, for the publication of any potentially identifiable images or data included in this article.

## Author Contributions

QK cared for the patient and designed the project. SY, CL, and LY collected clinical information. HY, ZN, HL, and LW helped with the analysis. All authors have read and accepted the manuscript.

## Funding

This work was supported by the Scientific Research Project of Hunan Health and Family Planning Commission (B20180528). Hunan Health and Family Planning Commission supervised this study.

## Conflict of Interest

The authors declare that the research was conducted in the absence of any commercial or financial relationships that could be construed as a potential conflict of interest.

## Publisher's Note

All claims expressed in this article are solely those of the authors and do not necessarily represent those of their affiliated organizations, or those of the publisher, the editors and the reviewers. Any product that may be evaluated in this article, or claim that may be made by its manufacturer, is not guaranteed or endorsed by the publisher.

## Abbreviations

SLC13A3: sodium-dependent dicarboxylate transporter member 3
NaDC3: Na+/Dicarboxylate Cotransporter 3
EEG: electroencephalogram
MRI: magnetic resonance imaging
WM: white matter
CSF: cerebrospinal fluid
NAA: N-acetylaspartate
WES: whole-exome sequencing.
